# OCTAVie: Open-source Capture Technology for Animal Vocalization in ultrasonic range

**DOI:** 10.1016/j.ohx.2026.e00771

**Published:** 2026-04-12

**Authors:** Laura Durieux, Jean-Baptiste Kammerer, Simon Schmitt, Denis Muller, Alexandra Barbelivien, Monique Majchrzak, François Stock, Frederic Antoni

**Affiliations:** a Université de Strasbourg, Centre National de La Recherche Scientifique, ICube Laboratory, UMR 7357, Strasbourg, France; b FabLab l’Atelier, Faculty of Physics and Engineering, Université de Strasbourg, Strasbourg, France; c Faculty of Physics and Engineering, Université de Strasbourg, Strasbourg, France; d Université de Strasbourg, Centre National de La Recherche Scientifique, Laboratoire de Neurosciences Cognitives Et Adaptatives (LNCA), UMR 7364, Strasbourg, France

**Keywords:** Ultrasonic vocalizations, Open-hardware, FPGA, Microphone, Real-time sonogram, Neuroscience

## Abstract

Ultrasonic vocalizations (USVs) in rodents are a key tool in neuroscience for investigating emotion, social behavior, and disease models. However, commercial recording systems remain expensive and generate massive datasets that require heavy post-processing. We present an open-hardware acquisition chain—from microphone to computer—designed to compute and display sonograms in near real time, thus reducing both storage needs and analysis workload. The system integrates (i) a custom ultrasonic microphone capable of detecting signals up to 100 kHz, (ii) a dedicated analog front-end with band-pass and anti-aliasing filtering, (iii) an Intel® Cyclone V GX FPGA implementing on-board Fast Fourier Transform processing and power calculation, and (iv) a lightweight software interface for data transfer and visualization. All design files, including Printed Circuit Board (PCB) layouts, VHDL codes, and C software, are openly released to ensure reproducibility. Compared to traditional microcontroller-based acquisitions, this architecture reduces raw data storage by more than 50% while maintaining a frequency resolution of ∼0.39 kHz and a temporal resolution of ∼2.5 ms − sufficient to resolve both 22 kHz and 50 kHz USVs. Validated with synthetic signals and experimental recordings, the platform provides neuroscientists with a low-cost, modular, and fully transparent tool for studying ultrasonic communication.

## Graphical abstract

**Figure:**
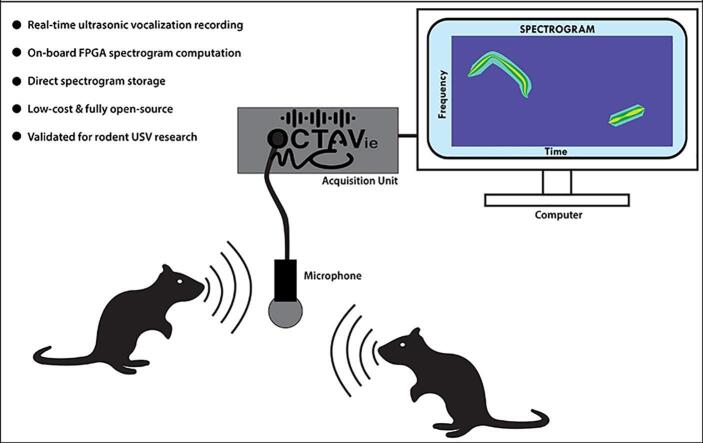

## 1 Specifications table

**Table:** 

Hardware name	OCTAV: Open-source Capture Technology for Animal Vocalization − Open-hardware ultrasonic vocalization acquisition system (microphone–FPGA–software chain)
Subject area	• *Neuroscience* • *Ethology*
Hardware type	• Measuring marker of behavior • Field measurements and sensors • *In-lab sensor*
Closest commercial analog	Ultrasonic rodent vocalization recording systems (e.g., Avisoft UltraSoundGate, Noldus UltraVox)
Open source license	Hardware: CERN Open Hardware License v2 (CERN OHL v2)Software: GNU General Public License version 3 (GPLv3)Documentation license: Creative Commons Attribution-ShareAlike 4.0 International (CC-BY-SA 4.0).
Cost of hardware	∼300–500 € (including microphone, analog filter PCB, FPGA development board, connectors, and assembly)
Source file repository	Uploaded on Zenodo: 10.5281/zenodo.17356089

## 2 Hardware in context

The study of ultrasonic vocalizations (USVs) in rodents is a widely used approach in neuroscience for probing social communication, emotional states, and neuropsychiatric models [1], [2], [3], [4], [5], [6]. USVs are extensively investigated in both rats and mice, although their frequency ranges and behavioral contexts differ partially between species [7], [8], [9]. In adult rats, vocalizations are typically categorized around 22 kHz and 50 kHz bands, while adult mice emit vocalizations within overlapping ultrasonic frequency ranges. Ultrasonic acoustic signals are also present in other vertebrate taxa. Echolocating bats produce high-frequency pulses that extend well into and beyond the ultrasonic range, serving for navigation and social communication across many species [10], [11]. In addition, certain frog species have been documented to emit vocalizations containing ultrasonic components [12], [13]. The specific frequency bands involved vary considerably between species. In the present work, OCTAVie was developed and experimentally validated using rat ultrasonic vocalizations. Despite its importance, the diffusion of USV research has been constrained by the high cost and closed nature of proprietary ultrasonic recording systems, which limits accessibility, reproducibility, and customization.

The OCTAVie platform addresses this challenge by providing a fully open-source, low-cost hardware chain designed specifically for neuroscience applications. Its architecture integrates a wide-band ultrasonic microphone and analog front-end with an Field Programmable Gate Array (FPGA)-based processing pipeline that computes Fast Fourier Transform (FFT) spectrograms in real time. Instead of storing raw ultrasonic waveforms—which require specialized post-processing to extract relevant features − OCTAVie directly outputs and records spectrograms. This shift substantially lowers storage requirements while making subsequent analysis more straightforward, since data can be handled as images rather than as high-frequency audio signals. While storing raw waveforms allows complete post hoc reanalysis with alternative signal-processing strategies, many ultrasonic vocalization studies ultimately rely on time–frequency representations derived from spectrograms. OCTAVie therefore prioritizes direct spectral storage to simplify routine workflows and reduce data volume, acknowledging this deliberate design trade-off.

The following table (Table 1) compares OCTAVie with representative commercial solutions used for rodent USV recordings. While commercial systems provide turnkey acquisition and proprietary workflows, OCTAVie uniquely combines an open hardware/software stack with on-FPGA spectrogram computation, enabling direct storage of spectrogram frames and improved reproducibility.

**Table 1:** Comparison between the OCTAVie system and representative commercial ultrasonic acquisition systems (Avisoft UltraSoundGate 116H and Noldus UltraVox), highlighting differences in openness, data architecture, processing approach, and cost.

	OCTAVie	Avisoft UltraSoundGate 116H	Noldus UltraVox
Openness/reproducibility	(CERN OHL v2/GPLv3/CC-BY-SA)	Proprietary	Proprietary
Spectrogram computation	On-FPGA (near real time)	PC-side software workflow	PC-side software workflow
Data architecture	On-board spectral transformation	Raw time-domain waveform stored	Raw time-domain waveform stored
Stored data volume (vs raw, same Fs and resolution)	∼50% reduction by spectral symmetry	100%	100%
Sampling rate (reported)	200 kHz	Up to 1000 kHz (selectable)	250 kHz default, up to ∼384 kHz reported depending on mic/version
Resolution	12-bit	16-bit or 8-bit (model dependent)	16-bit
Channels	1 channel (dual-input subtraction implemented)	1 channel	1 channel (per device)
Cost (transparent)	∼300–500 **€** (BOM provided)	5000 € (unit)/6000 € (kit with mic)	Quote-based (pricing on request)

By lowering both economic and technical barriers, OCTAVie enables a broader range of laboratories to include ultrasonic communication in their behavioral studies. Its open-source nature ensures transparency, encourages community-driven improvements, and facilitates integration with existing open analysis ecosystems, including machine learning–based detectors [14]. In this way, the hardware contributes to more accessible, reproducible, and flexible neuroscience research.

## 3 Hardware description

### 3.1 Overview

The OCTAVie platform is organized as a modular acquisition chain dedicated to the study of ultrasonic vocalizations (USVs) in rodents. The system is divided into four functional levels that reflect the progression of the signal from capture to analysis:

• Microphone – captures vocalizations in the 10–100 kHz range.
• Acquisition unit – composed of two submodules:
  o Analog signal board, responsible for amplification, band-pass filtering, and gain adjustment,
  o FPGA development kit, integrating the Analog to Digital Converter (ADC) for digitization and managing the data pipeline.
• FPGA firmware – performs continuous time-based spectral analysis, magnitude computation, and data formatting for transmission.
• Software – running on a host computer, receives formatted data from the FPGA, synchronizes frames, and stores spectrograms in a structured format suitable for subsequent analysis.

This modular organization allows each block to be developed and validated independently while maintaining a coherent overall architecture. The microphone provides the raw ultrasonic signal, the analog board conditions for digitization, the FPGA executes the computationally intensive transformations in real time, and the host software manages visualization and storage.

**Fig. 1:**
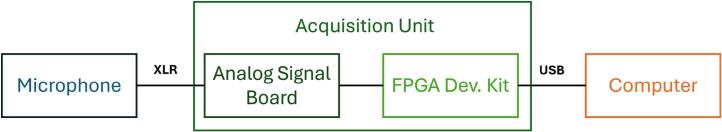
General block diagram of the OCTAVie system.

The signal path is illustrated in Fig. 1, which presents the high-level architecture and the data flow through the four main modules. Detailed schematics of the analog signal board, FPGA internal structure, and software flow will be provided in the corresponding sections.

In electronic system design, component lifecycle evolution is an inherent constraint that must be anticipated. The OCTAVie platform was therefore developed with a modular and adaptable architecture, allowing equivalent components to be substituted when required without affecting the overall acquisition and processing chain. The fully open-source documentation ensures that such updates can be implemented transparently, supporting long-term sustainability and reproducibility of the system.

### 3.2 Microphone

The microphone module is based on a wide-band MEMS ultrasonic transducer (SPU0410LR5H-QB, Knowles), chosen for its ability to cover the frequency range of interest for rodent vocalizations. The manufacturer specifies a relatively flat response up to 80 kHz, with measurable sensitivity extending close to 100 kHz, an omnidirectional pattern, a sensitivity of –38 dBV/Pa, an impedance of ∼400 Ω at 1 kHz, and a signal-to-noise ratio of 63 dB(A). The package dimensions (3.76 × 3.00 × 1.10 mm) enable integration on a PCB.

Because the raw microphone output is in the order of microvolts, a preamplification stage is mandatory. We evaluated several operational amplifiers and retained the MCP6021, which offers a high gain-bandwidth product and maintains a flat response across the ultrasonic band of interest. The amplifier was configured for a gain of 50, which provides sufficient output amplitude while avoiding saturation when connected to the subsequent acquisition chain. This gain corresponds to the fixed preamplification stage on the microphone PCB and is distinct from the additional adjustable gain stages implemented on the analog signal board described in the following subsection.

The module is powered from a single 5 V supply rail used across the analog chain. Since the MEMS capsule operates at 3.3 V, a resistive voltage divider was added to derive a stable bias from the 5 V line. A status LED is integrated on the PCB to indicate when the microphone is powered, an important feature for experimental setups where multiple units may be deployed simultaneously. Signal and power are routed through a three-pin XLR connector, which ensures robustness and shielding against external noise. In our design, one pin carries the amplified audio signal, one provides the ground reference, and the third is repurposed to deliver power to the microphone board. This choice of professional-grade connector simplifies integration into laboratory setups, while maintaining mechanical robustness.

**Fig. 2:**
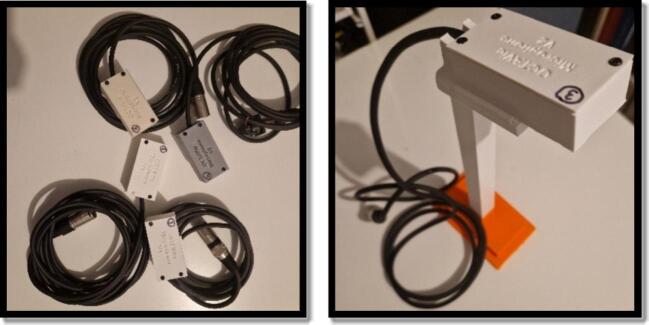
On the left, photograph of the microphone circuit into its enclosure, with this XLR connector (x4). On the right, photograph of the microphone on its holder.

The assembled PCB includes the MEMS capsule, the biasing network, the preamplifier, the status LED, and the XLR interface. A 3D-printed housing (Fig. 2) was developed to protect the circuit while leaving the acoustic port unobstructed, allowing reliable deployment in behavioral cages. We have also included a 3D printed design of a microphone holder to allow placement on top of the housing cage, with the microphone directed towards the animals. All files required to reproduce the microphone are available in the repository under \2.hardware\1.microphone\v1.0.

### 3.3 Acquisition unit

**Fig. 3:**
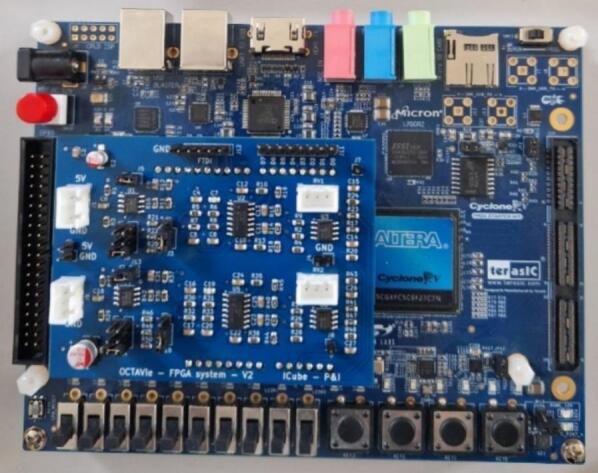
FPGA development board and Analog Signal Board assembly.

The acquisition unit is responsible for conditioning the microphone signal, digitizing it at high resolution, and transferring it to the FPGA for spectral analysis. It is composed of two modules: a custom-designed analog signal board and a commercial FPGA development kit with integrated ADC (Fig. 3). All design and source files necessary to reproduce the acquisition unit are located in the repository at OCTAVie\2.hardware\2.acquisition_unit\v2.0.

#### 3.3.1 Analog signal board

**Fig. 4:**
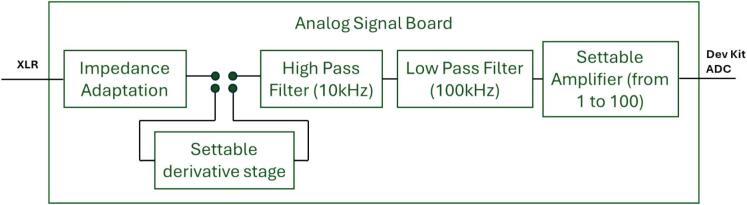
Diagram of signal processing of the analog signal board.

The analog front-end prepares the weak preamplified microphone signal for digitization. Its design combines amplification, filtering, and gain adjustment, ensuring that the signal is matched to the input dynamic range of the ADC (Fig. 4). The main functions are as follows:

• Impedance adaptation: The board ensures proper impedance matching between the microphone/preamplifier stage and the ADC input.
• Derivative stage: To enhance sensitivity to the higher part of the ultrasonic spectrum, the board integrates a derivative block that increases gain with frequency. The slope of this frequency-dependent amplification can be configured by jumpers with three selectable positions, allowing the user to adapt the emphasis on high-frequency content. If not required, the entire derivative stage can be bypassed using dedicated jumpers, ensuring flexibility of operation.
• Band-pass filtering: The signal path includes a cascaded high-pass filter with a cutoff frequency around 10 kHz, attenuating low-frequency environmental noise, and a low-pass filter at 100 kHz to suppress high-frequency components above the Nyquist limit. The topology is based on active second-order filters using low-noise op-amps.
• Amplification: A low-noise operational amplifier (MCP6021) provides adjustable gain stages. The board can operate at different gain levels (×1, ×10, ×100) settled by a potentiometer. This flexibility allows adaptation to different experimental conditions, such as close-range recordings with high Signal-to-Noise Ratio or long-range detection with lower input amplitudes. These gains are applied after the fixed preamplification stage of the microphone module.
• Power supply and connectivity: The analog board operates from a 5 V line and is connected to the microphone via a shielded 3-pin XLR cable. The conditioned signal is sent to the FPGA development kit through standard pin headers.

The PCB was implemented on a four-layer board. The layout was optimized to minimize parasitic capacitance and inductance, both critical for preserving ultrasonic fidelity. This was achieved through the use of a continuous ground plane, short and direct routing of high-frequency signal traces, minimized loop areas, and careful separation between analog signal paths and power lines. Critical components were placed in close proximity to reduce trace length and associated parasitic effects.

#### 3.3.2 FPGA development kit

The digitization and digital processing stages are handled by a Terasic Cyclone V GX Starter Kit, chosen for its balance of cost, flexibility, and compatibility with open development tools. The board integrates:

• ADC interface: An LTC2308 12-bit successive approximations ADC provides sampling rates up to 200 kS/s across multiple channels. The ADC is controlled via Serial Peripheral Interface (SPI), and its timing is managed directly by the FPGA. At 200 kS/s, the system achieves a frequency resolution of ∼ 0.39 kHz with a 512-point FFT, which is suitable for distinguishing between typical 22 kHz and 50 kHz calls.
• FPGA: The Cyclone V GX (5CGXFC5C6F23C7N) provides sufficient logic resources to implement the FFT pipeline, memory buffers, and communication blocks.
• Connectivity: The FPGA board communicates with the host computer via a USB-to-Universal Asynchronous Receiver-Transmitter (UART) bridge (FTDI chip). Data are transmitted as structured frames at a sustained throughput up to 3 Mbaud, which is compatible with continuous spectrogram generation in real time.
• Power and clock management: The board is powered through its own power supply and distributes regulated rails to both the ADC and the FPGA. This power is also distributed to the analog frontend board (microphone and analog signal processing board). A clock divider within the FPGA derives the precise timing needed for ADC sampling and FFT computation.

### 3.4 FPGA firmware

**Fig. 5:**
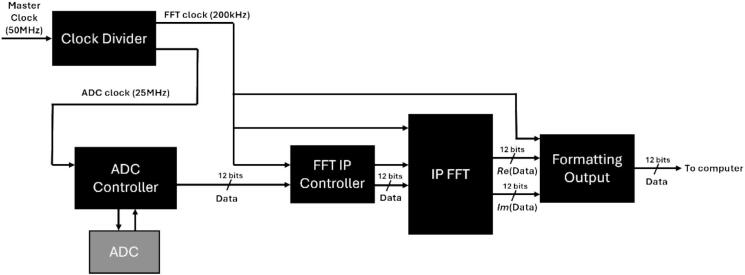
Schema of principle of the different block organization. The grey box is just an indication and represents the component directly.

The FPGA firmware implements a streaming pipeline that converts the digitized ultrasonic signals into power-spectral frames suitable for immediate storage and visualization. The design is entirely written in VHDL, and all source code is openly available, except for the FFT core and arithmetic blocks which rely on proprietary Intel IP; access to and modification of these IP components remains at the discretion of users who choose to acquire the corresponding licenses. Nevertheless, the compiled bitstream of theses proprietary IP can be freely distributed. The firmware architecture is organized as a sequence of dedicated modules: clock generation and distribution, ADC control, dual-microphone subtraction, FFT computation, spectral power calculation, half-spectrum selection, and UART frame formatting for data transfer. The system clock on the Cyclone V board runs at 50 MHz, from which derived clocks are generated for the ADC SPI interface, the FFT streaming pipeline, and the 3 Mbaud serial output link. In the verified implementation, the ADC domain operates at 25 MHz, the acquisition and FFT pipeline process samples at 200 kS/s, and a phase-locked loop produces an accurate 3 MHz clock for the UART, ensuring reliable high-throughput data transmission. The design (Fig. 5) was developed and compiled using Intel Quartus Prime Lite Edition 19.1 (2019), which provides synthesis, place-and-route, and IP integration tools specifically for Cyclone V devices (Intel Corporation). The FPGA firmware sources, including all VHDL code, are available in the repository under OCTAVie\3.firmware_fpga\1.vhdl. The corresponding compiled bitstream files can be found in OCTAVie\3.firmware_fpga\2.builds.

#### 3.4.1 ADC control

The interface with the LTC2308 12-bit Successive Approximation Register ADC is handled entirely inside the FPGA by a dedicated SPI controller that ensures deterministic timing at the required 200 kS/s acquisition rate. At each cycle, the controller asserts the chip-select line, shifts out the 4-bit command word on MOSI to configure the converter, and then drives 12 SCLK pulses to capture the digital output word bit-by-bit on MISO, starting with the most significant bit. The two input channels are acquired in alternation at the configured sampling rate, resulting in two time-aligned 200 kS/s sample streams (one per channel). A subtraction stage immediately computes the difference between these channels (sA – sB). The two ADC inputs correspond to two independent analog input paths on the acquisition board, allowing two microphones to be connected simultaneously. In normal operation, one microphone may serve as a reference for noise cancellation, and in the absence of a connected sensor effectively removes the static offset generated by the analog electronics. Each completed 12-bit word is transferred into a shift register, flagged as valid, and pushed into a FIFO buffer that decouples the SPI domain from the downstream FFT pipeline. The full sequence of command, conversion, and readout takes 5 μs, matching the throughput required for continuous ultrasonic acquisition.

#### 3.4.2 FFT processing

The spectral decomposition of the acquired signal is performed inside the FPGA using the Intel FFT IP core configured for 512-point streaming operation. A dedicated control block manages the handshake signals required by the core (sop/eop, valid, ready), ensuring that one frame of 512 samples is accumulated and processed without interruption. The incoming stream from the FIFO is reformatted into the real-input interface of the FFT (imaginary part fixed to zero) and synchronized so that each sample is applied on a valid clock edge. Once the transformation is initiated, the FFT core produces complex outputs in natural order, aligned to the same streaming protocol. The control logic tracks the start and end of each block to maintain a precise correspondence between 512 input samples and 512 frequency-domain bins. With the system configured at a sampling frequency of 200 kS/s, this implementation yields a spectral resolution of approximately 0.39 kHz and a temporal resolution of 2.56 ms per frame, which matches the requirements for analyzing both short 50-kHz calls and longer 22-kHz calls in rodents. The deterministic timing of the FFT IP guarantees that each frame is delivered at a fixed latency, enabling continuous operation of the pipeline.

#### 3.4.3 Power calculation

Following the FFT stage, the complex output for each frequency bin is converted into a power estimate by a dedicated hardware block inside the FPGA. The real and imaginary components, both delivered in fixed-point format from the FFT IP, are squared in parallel using two multiplier IPs, producing 24-bit intermediate results. These values are then summed by a pipelined adder to compute the squared magnitude according to P = Re^2^ + Im^2^. Since all values are non-negative after squaring, the datapath is optimized to operate without overflow using 24-bit registers, which provide sufficient dynamic range for the ultrasonic signals of interest. The computation is performed in a streaming manner: each FFT bin is processed as soon as it is available, with no additional buffering required. The resulting power values form a sequence of 512 bins per frame, with deterministic latency from input to output.

#### 3.4.4 Buffering

Only the first 256 FFT bins corresponding to the 0–100 kHz range (i.e., the non-redundant half of the spectrum for a 200 kS/s sampling rate) are retained, while the symmetric half is discarded. This operation does not reduce the acquisition sampling frequency, which remains 200 kS/s, but only limits the stored spectral data to the physically meaningful positive-frequency range. Only the first 256 bins (0 → Fs/2) are retained; the symmetric half is dropped. A 256-entry on-chip memory captures one bin per FFT output clock, then reads out at half-rate (one value every two clock cycle), thereby reducing the internal spectral data throughput by a factor of two for the downstream serializer, without affecting the 200 kS/s time-domain sampling frequency. The buffer is aligned using the FFT source_sop to ensure exact frame boundaries. Since the FFT produces 512 bins per frame but only the positive frequency components are relevant for spectral analysis, the firmware retains the first 256 bins corresponding to the 0–100 kHz band and discards the symmetric half. To achieve this, the power values are written sequentially into a dual-port block RAM that acts as a circular buffer, with the write pointer synchronized to the FFT start-of-packet signal. Once a frame is complete, the read pointer begins extracting the stored bins at half the internal clock rate, effectively downshifting the data stream from the 200 kHz FFT output domain to a 100 kHz serialization domain compatible with the UART transmitter. This scheme ensures that exactly one power value is output every two clock cycles, maintaining alignment between successive frames while easing timing closure for the high-speed serial interface. The buffering also provides deterministic framing: each block of 256 bins is clearly delimited by the control logic, which asserts a synchronization flag to the formatter at the beginning of each readout.

#### 3.4.5 UART communication

**Fig. 6:**
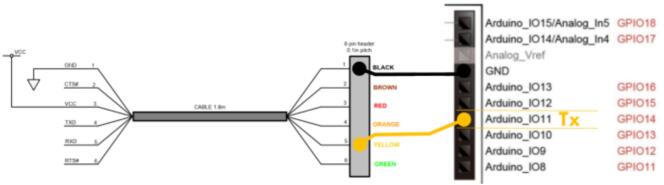
FTDI pinout for our data transfer. Adapted from both Cyclone V GX and FTDI datasheets.

**Fig. 7:**
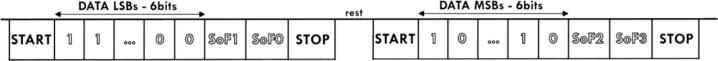
Format of the UART frames sent to the computer. SoFs bits are at ‘1′ when a start of FFT frame is detected.

Once the 256-bin half-spectrum has been computed and buffered, the final stage of the FPGA firmware formats the data into serial frames for transfer to the host computer via a USB-UART bridge operating at 3 Mbaud (Fig. 6). Each spectral bin is represented as a 12-bit fixed-point value. To transmit these values efficiently, the formatter splits each word into two bytes: the six most significant bits are stored in the lower part of the first byte, and the six least significant bits in the lower part of the second byte. The two upper bits (bits 7 and 6) of each byte are reserved as synchronization flags. For every new FFT frame, these two bits are both set to logic ‘1′ in the first transmitted pair, creating a unique “start-of-frame” (SoF) marker that allows the host software to unambiguously identify the beginning of each spectrogram. For all subsequent bins, these flag bits are cleared to zero, ensuring that in the data stream only the frame headers contain the synchronization signature (Fig. 7). The UART logic is clocked by a dedicated Phase-Locked Loop that produces an accurate 3 MHz baud clock from the 50 MHz system oscillator, guaranteeing bit-level alignment across long recordings. A finite-state machine handles byte serialization, inserting start and stop bits as according to the UART protocol, and schedules the transmission of exactly 512 bytes (256 bins × 2 bytes) per frame. The formatter thus ensures that each spectrogram frame is delivered in a deterministic sequence, fully synchronized to the FFT cycle, and ready for direct reconstruction on the host computer.

### 3.5 Software

The OCTAVie software is a desktop application written in C and built on the GTK4 framework. Its primary purpose is to acquire real-time spectral data transmitted from the FPGA board, visualize it as a two-dimensional spectrogram, and optionally record it to disk for further analysis. The program has been designed with a modular architecture, in which the graphical user interface (GUI), communication routines, and data-handling threads are separated into distinct modules (gui.c/.h, io.c/.h, main.c, and msg.h). The complete PC software source code is available in the repository under OCTAVie\4.software_pc\1.sources, while the compiled executables are located in OCTAVie\4.software_pc\2.build.

#### 3.5.1 Graphical user interface

**Fig. 8:**
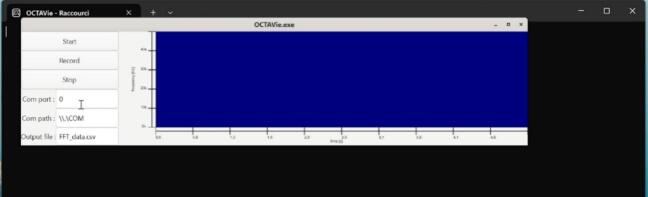
Graphical interface of OCTAVie.exe.

The GUI module (gui.c, gui.h), Fig. 8, defines the application window and the interactive elements that allow users to control the acquisition. The main window is organized into three panels:

• a central spectrogram display (GtkPicture), updated continuously by the rendering thread,
• one drawing area dedicated to the axes, showing elapsed time along the horizontal axis and frequency along the vertical axis,
• and a control panel on the left with three buttons (“Start”, “Record”, “Stop”) and text fields for selecting the COM port, the port path, and the output filename.

Color rendering of the spectrogram is handled through a dedicated value_to_color() mapping function, which scales raw FFT amplitudes logarithmically and assigns a color gradient (blue-green–red) to represent increasing magnitudes.

#### 3.5.2 Data acquisition and communication

The acquisition routines are implemented in the io.c/io.h module. Communication with the FPGA is performed via a serial COM port at 3 Mbaud. The code provides compatibility for both POSIX (Linux) and Windows environments, although the primary target platform is Linux.

The read_com() thread continuously listens to the serial interface, synchronizes on frame headers, and decodes incoming data packets into two alternating memory buffers. Synchronization recovery routines are implemented to mitigate data corruption or transmission errors. The design ensures that data loss is explicitly detected and logged, while blank frames are substituted during re-synchronization to preserve temporal continuity.

#### 3.5.3 Multithreading and data management

The software relies extensively on POSIX threads (pthread) for concurrent execution. Three persistent worker threads are created at startup:

• Communication thread (read_com): manages serial input and buffer filling.
• Recording thread (record_data): writes buffered samples to a.csv file, including a time stamp for each frame.
• Graph update thread (update_graph): maps the most recent buffer content to the spectrogram display.

Thread synchronization is ensured by condition variables and mutexes, which coordinate buffer handovers between producer and consumer threads. The shared state of the application is maintained in the AppState structure, which contains synchronization primitives, buffer pointers, and GUI element references.

#### 3.5.4 File output

When activated, the recording thread stores the acquired spectra into a comma-separated values (CSV) file (default: FFT_data.csv). Each line of the file corresponds to one frame and begins with an absolute time stamp, followed by the spectrum values. This format facilitates direct use in external environments such as Python, MATLAB, or spreadsheet applications for post-processing.

#### 3.5.5 Program execution flow

The entry point (main.c) initializes the shared state, allocates memory buffers, and creates the three worker threads. It then launches a GtkApplication that manages the event loop and user interaction. On exit, the application gracefully terminates the worker threads, releases allocated resources, and closes the output file if necessary.

#### 3.5.6 Portability and build

The software can be compiled with a standard makefile, provided that the GTK4 development libraries are available. Definning symbolic constants into the makefile (OS_POSIX or OS_WIN) allows compilation under Linux or Windows.

### 3.6 Key specifications

The OCTAVie system acquires ultrasonic vocalizations in the 10–100 kHz range using a Micro-Electro-Mechanical Systems (MEMS)-based microphone with dedicated preamplification, an analog conditioning board with a band-pass of ∼10–100 kHz, and a dual-channel 12-bit Successive Approximation Register ADC operating at 200 kS/s. In summary, the ADC sampling frequency is 200 kS/s, resulting in a Nyquist frequency of 100 kHz. The retained 0–100 kHz band therefore corresponds to the non-redundant half-spectrum of the real-valued input signal. The digital processing chain is implemented on a Cyclone V FPGA running a 512-point streaming FFT, providing a frequency resolution of ∼0.39 kHz and a temporal resolution of ∼2.6 ms per frame. Only the positive half of the spectrum (256 bins) is retained, converted into power values, and serialized at 3 Mbaud via an USB-UART link. The host software receives the frames, reconstructs the 12-bit bins, and stores them as CSV tables where rows correspond to time frames and columns to frequency bins. The total latency from acquisition to storage is deterministic and equal to one FFT frame (∼2.6 ms). The microphone module consumes less than 2 mA at 5 V, while the FPGA board operates from its standard supply, keeping the overall system power low and compatible with extended recording sessions.

Key advantages:

• Low cost and open source: the complete design (hardware, VHDL firmware, and software) is openly available, reducing economic and technical barriers compared to proprietary ultrasonic systems.
• Simplified data analysis: spectrograms are generated and stored directly, allowing researchers to work with image-like data rather than raw high-frequency waveforms.
• Customizable architecture: each stage (microphone, analog board, FPGA firmware, host software) can be adapted or extended for specific research needs.
• Scalable design: the dual-channel acquisition allows noise cancellation or multi-microphone setups, and the modular pipeline supports further developments such as on-FPGA classifiers.
• Reproducibility and collaboration: the openly documented system encourages validation, improvement, and adoption by a broad neuroscience community.

## 4 Potential applications and broader use

• Enables real-time monitoring of ultrasonic vocalizations without requiring post-processing of raw audio data, facilitating rapid experimental feedback and reducing analysis workload.
• Provides a low-cost and fully open-source alternative to proprietary systems, allowing laboratories with limited resources to implement ultrasonic recording capabilities.
• Supports flexible experimental configurations, including noise subtraction using dual-channel acquisition or adaptation of the analog and digital processing chain for different frequency ranges or species.
• Allows integration with automated analysis pipelines, including machine learning-based detectors, by directly generating structured spectrogram data suitable for downstream processing.
• Can be repurposed for other high-frequency sensing applications (e.g., bioacoustics, environmental monitoring, or ultrasonic signal characterization) beyond the initial neuroscience use case.

## 5 Design files summary

All the required information regarding the design files — including file names, types, open-source licenses, and storage locations — are compiled in the document File_list_v1.0.xlsx, located at the root of the repository. Each design file associated with the hardware, including all primary and secondary sources, is listed in a separate row as requested. Given that the complete table contains nearly one hundred entries, it was not included in the main text to maintain readability. A summary table has, however, been added below (Table 2).

**Table 2:** Summary of design files included in the OCTAVie project.

**Design file name**	**Content description**	**File type**	**Open source license**	**Location of the file**
*Documentation*	*User guides, build instructions, programming tutorials, PCB design guides, system documentation*	*PDF, DOCX*	*CC BY-SA 4.0*	Uploaded on Zenodo: 10.5281/zenodo.17356089
*Microphone hardware*	PCB design files, fabrication files (Gerber, drill), BOMs, assembly files	KiCad, GBR, DRL, XLSX, PDF	CERN OHL v2	Uploaded on Zenodo: 10.5281/zenodo.17356089
Acquisition unit hardware	Analog signal board design, fabrication files, BOMs, FPGA devkit integration	KiCad, GBR, DRL, CSV, XLSX	CERN OHL v2	Uploaded on Zenodo: 10.5281/zenodo.17356089
Mechanical parts	Enclosures and mechanical components for microphone and acquisition unit	3MF CAD files	CERN OHL v2	Uploaded on Zenodo: 10.5281/zenodo.17356089
Firmware (FPGA)	VHDL source files, FFT processing, ADC control, communication modules, compiled bitstream	VHDL, POF	GPLv3	Uploaded on Zenodo: 10.5281/zenodo.17356089
Software (PC application)	GUI, data acquisition interface, processing and visualization software	C (source + build)	GPLv3	Uploaded on Zenodo: 10.5281/zenodo.17356089
Data & examples	Example FFT data format and real recording datasets	CSV, ZIP	CC BY-SA 4.0	Uploaded on Zenodo: 10.5281/zenodo.17356089
Validation & test protocols	Experimental procedures for validating microphone, acquisition unit and full system	PDF, DOCX	CC BY-SA 4.0	Uploaded on Zenodo: 10.5281/zenodo.17356089
Release packages	Ready-to-use fabrication files and compiled binaries	ZIP, POF	CERN OHL v2/GPLv3	Uploaded on Zenodo: 10.5281/zenodo.17356089
Project *meta*-files	Licenses, citation file, contribution guidelines	TXT, MD, CFF		Uploaded on Zenodo: 10.5281/zenodo.17356089

## 6 Bill of materials summary

To ensure readability and clarity within the main text, only the high-level structure of the bill of materials (BOM) is included in the article (Table 3). The full detailed BOM, containing individual component references, unit costs, suppliers, and material types, is provided as a supplementary file. This approach prevents the article from being overloaded with tabular data while still guaranteeing full transparency and reproducibility of the system. Readers interested in the complete cost breakdown and sourcing information can easily access it in the repository at \1.docs\1.overview\BOM_full_v1.0.xlsx.

**Table 3:** High level BOM and cost estimation for the OCTAVie system, highlighting component sourcing, subsystem contributions, and overall system cost.

Designator	Number	Cost per unit (€)	Source of materials	Material type
**Microphone**				
Electronic component	1	5,24	Digikey	Other
PCBA	1	24,35	JLCPCB	Other
System assembly	1	55,06	Digikey	Other
Enclosure	1	61,14	JLCPCB	Other

**Analog Signal Board**				
Electronic component	1	20,91	Digikey	Other
PCBA	1	24,35	JLCPCB	Other

**FPGA**				
Electronic component	1	264,63	Mouser	Other

**System**				
System assembly	1	23,44	Digikey	Other
Enclosure	1	36,87	JLCPCB	Other
**Total**				
		**515,99**		

## 7 Build instructions

The complete construction instructions for all three hardware modules are available in the repository under \1.docs\2.build_guide. Each section provides detailed, step-by-step assembly guidance, complemented by images and schematics. Additionally, module-specific build instructions are included within the corresponding subfolders of the hardware directory, ensuring that each component can be independently reproduced and assembled.

### 7.1 Microphone

After receiving the fabricated PCB from JLCPCB, the board is separated from the panel along the V-cuts and inserted into the custom 3D-printed enclosure. The microphone opening is aligned with the front aperture and fixed using four M2 screws. A 2 m shielded XLR cable is then prepared: the outer sheath and internal wires are stripped, and the three conductors are soldered to the XLR male connector following the standard pinout—left pin to ground (shield), middle pin to signal, and right pin to power.

At the other end of the cable, JST-PH terminals are crimped and inserted into a 3-pin female connector corresponding to the PCB pinout (Power, GND, Signal). The cable is routed through the side opening of the enclosure and connected to the microphone board before closing the lid with screws. Continuity checks are performed to ensure proper wiring and absence of short circuits. Once verified, the microphone is ready for operation.

### 7.2 Acquisition unit

The construction of the acquisition system of OCTAVie system involves three main steps: preparation of the analog signal PCB, programming of the FPGA, and mechanical/electrical assembly into the custom 3D-printed enclosure.

1) Analog Signal PCB – Upon delivery, users must configure the derivative stage by setting jumpers (J3/J10, J6/J14). These adjustments allow tailoring the frequency response to experimental conditions.
2) FPGA Programming – The Cyclone V board is flashed with a.pof configuration file using Quartus Prime Lite. After switching to programming mode, the.pof is loaded in Active Serial Programming mode via USB-Blaster. Once programmed, the board is switched back to “Run” and validated through test signals. This step ensures reproducibility of the open FPGA firmware.
3) Enclosure Assembly – The FPGA and analog daughterboard are mounted into the 3D-printed base using standoffs and M3 screws. Potentiometers are wired via JST connectors, XLR outputs are soldered and mounted, and an FTDI cable is prepared to connect to the board header. All parts are then neatly routed before final closure of the enclosure.

Design decisions and alternatives:

- The derivative stage was made optional to provide flexibility in frequency emphasis.
- Standard JST and XLR connectors were chosen to ensure compatibility and safe handling.
- A 3D-printed enclosure was selected for low-cost prototyping; metal enclosures were considered but rejected due to cost and fabrication time.

Safety considerations:

- Ensure correct wiring of the XLR connector to prevent microphone damage.
- Do not disconnect the FPGA during programming.

Full, step-by-step illustrated tutorials (including schematics and images), as well as all design files and BOM references, are available in the Zenodo project repository.

### 7.3 Final step

Once assembly is complete, connect the microphone to the XLR plug, the power cable to the FPGA, and the USB cable to the computer. At this stage, the hardware is fully operational and ready to perform ultrasonic vocalization recordings.

## 8 Operation instructions

The OCTAVie system enables the real-time acquisition, processing, and visualization of ultrasonic vocalizations. Its operation follows a straightforward sequence ensuring safe and reproducible use. The complete, illustrated operating manual − including screenshots and troubleshooting examples − is available in the repository at \1.docs\3.user_guide.

### 8.1 System setup

Connect the FPGA box, microphones, and power supply as shown on the enclosure. Channel 1 and 2 microphones are connected to the two XLR ports at the rear. The USB FTDI cable links the FPGA box to the computer, and power is supplied through the dedicated connector. Once the top power button is pressed, the internal LED indicates initialization.

### 8.2 Software launch and configuration

Open the OCTAVie software on the computer. After startup, identify the correct COM port through the Windows Device Manager in Windows environment or thanks to the “usb-devices” command in POSIX environment, and enter its number in the interface. Define an output filename ending in “.csv”. To prevent accidental overwriting of existing recordings, the software automatically appends a timestamp to each file name at the start of a recording session. This ensures that every acquisition is stored as a unique file, even if the user does not manually modify the base filename. The interface allows real-time display of the incoming FFT frames.

### 8.3 Data visualization and recording

Press Start to visualize live ultrasonic spectrograms. To verify signal reception, snap fingers or produce a short metallic sound near the microphone—the response should appear as a localized pattern on the time–frequency display. When the signal amplitude is satisfactory, click Record to start data acquisition, then Stop to terminate it. The resulting CSV file can be opened directly in Excel; rows correspond to time steps (2.56 ms) and columns to frequency bins (390 Hz).

### 8.4 Signal adjustment

Recording sensitivity is controlled through the two rotary gain knobs on the front panel or the “+” and “–” push buttons on the FPGA board. These modify the amplification factor to optimize the signal-to-noise ratio. A reset button restarts the firmware if unexpected FFT behavior occurs. Advanced analog filtering options are available via internal jumper settings for experienced users.

## 9 Validation and characterization

### 9.1 Microphone

**Fig. 9:**
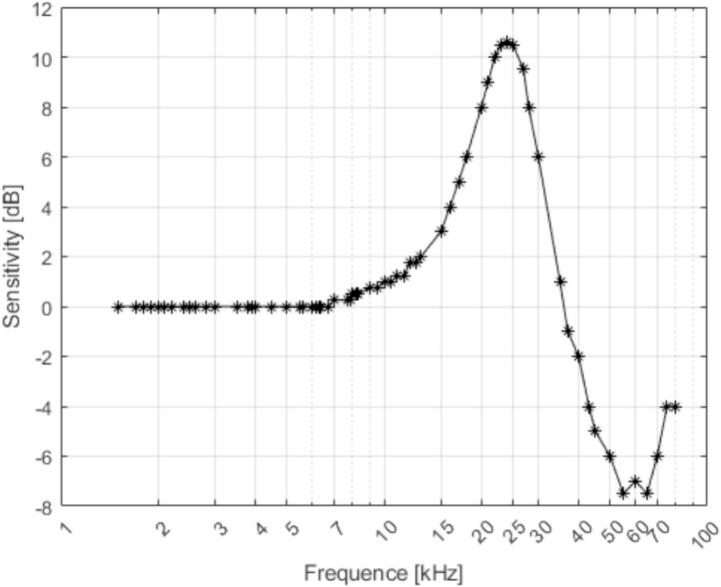
Bandwidth of the SPU0410LR5H-QB microphone, values taken from the manufacturer’s datasheet.

Validation experiments demonstrated that the microphone–amplifier module of OCTAVie exhibits the expected wideband ultrasonic performance. Characterization of the SPU0410LR5H-QB sensor, integrated with a low-noise operational amplifier (MCP6021), confirmed a usable frequency response from approximately 1.5 kHz up to nearly 100 kHz. These results are consistent with manufacturer specifications and were validated both on breadboard and on PCB implementations (Fig. 9).

In controlled tests, the analog stage consumed ∼1.5 mA at 5 V supply, a negligible load relative to the global system requirements. Long-term stability tests confirmed that no drift in sensitivity or frequency response was detectable after 18 h of continuous operation.

Capabilities and limitations of the microphone module:

• Frequency response: linear from 1.5 kHz to ∼100 kHz, covering the full range of rat USVs.
• Gain and noise: adequate signal-to-noise ratio after integrated pre-amplification; higher linearity than some commercial references.
• Linearity: detailed validation not available, but not required for this application since the aim is detection of vocalizations rather than precise acoustic quantification.
• Power consumption: ∼1.5 mA at 5 V, negligible in the overall system budget.
• Stability: no observable drift after 18 h of continuous operation.
• Durability: robust performance under repeated handling and extended operation.
• Limitations: characterization constrained by non-calibrated loudspeakers; absolute sensitivity remains to be established under standardized conditions.

### 9.2 Analog signal circuit

Validation of the analog anti-aliasing filter was carried out through a series of breadboard and PCB experiments. The objective of this stage is to restrict the signal bandwidth to 10–100 kHz, corresponding to the range of rat ultrasonic vocalizations and the Nyquist frequency imposed by the 200 kS/s sampling rate, while simultaneously providing gain adjustment to match the dynamic range of the ADC.

**Fig. 10:**
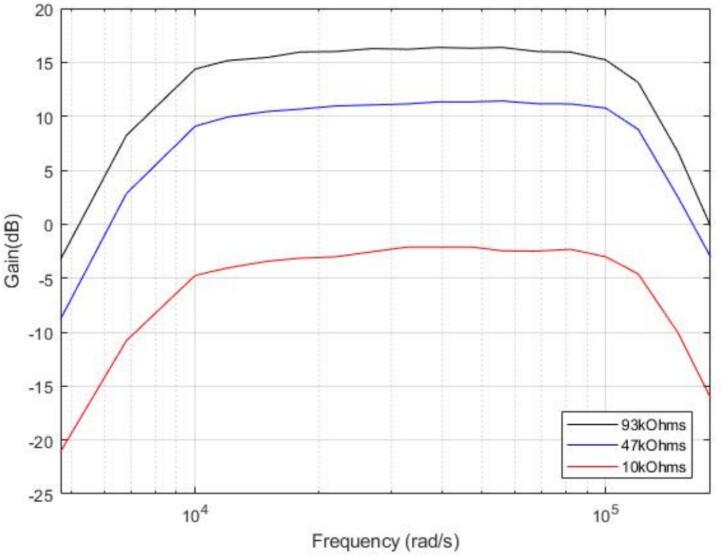
Bode results of the filter on the SMD version of the PCB for several gain (potentiometer setting).

The design was then fabricated as a surface-mount PCB. Bode measurements performed on the assembled circuit confirmed the expected behavior: the amplitude response was flat across the defined 10–100 kHz band, while frequencies below 10 kHz and above 100 kHz were strongly attenuated. Tests performed at different amplifier settings using the onboard potentiometer further confirmed that gain could be adjusted without distorting the band-pass characteristics (Fig. 10).

The final validation step demonstrated that the circuit meets all requirements: it reliably suppresses irrelevant frequency components, preserves the frequency content of ultrasonic vocalizations, and constrains the signal to the amplitude window required by the ADC. These results confirm that the analog signal conditioning stage performs its role as an anti-aliasing and amplification block, enabling accurate digitization of ultrasonic vocalizations for further FPGA processing.

Capabilities and limitations of the analog signal circuit:

• Band-pass response from ∼ 10 to 100 kHz, verified by Bode measurements.
• Adjustable amplification via potentiometer, without distortion of the filter response.
• Correct attenuation of out-of-band signals, both below 10 kHz and above 100 kHz.

### 9.3 FPGA acquisition

**Fig. 11:**
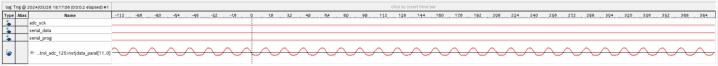
Representative ADC output captured via SignalTap during functional validation of the acquisition stage.

Validation of the FPGA stage focused on verifying the correct acquisition of the analog signal through the on-board ADC and the subsequent computation of the fast Fourier transform (FFT) using the Intel IP core. It is important to note that every stage of the FPGA design was validated either through testbench simulations or by using SignalTap, the built-in FPGA debugging tool that allows real-time visualization of internal signals. The tests confirmed that the ADC controller, implemented in VHDL, was able to generate the required SPI control signals and capture digitized data at the intended sampling rate of 200 kS/s. SignalTap measurements on the Cyclone V board showed consistent conversion of sinusoidal inputs, with results coherent with the expected digital waveform (Fig. 11). The slight waveform clipping visible in the sinusoidal test signal results from the characteristics of the non-calibrated signal source used during functional validation. This did not affect the verification of ADC timing, frequency localization, or FFT processing behavior, which were the primary objectives of this test.

**Fig. 12:**
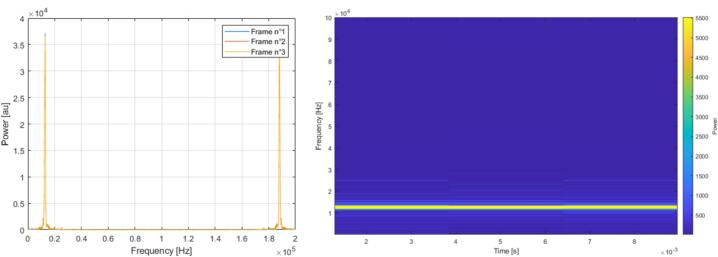
Example FFT spectrum obtained from a sinusoidal input during spectral validation of the FPGA processing chain. SignalTap exported data, showing the superposition of three perfectly matching frames of 12 kHz (on the left). Spectrogram of the 12 kHz frequency sinusoid signal from the hardware FFT (on the ritgh).

The FFT IP block was configured with 512 points, corresponding to a compromise between frequency and time resolution. This configuration provided a frequency resolution of approximately 390 Hz and a time resolution of 2.56 ms, which is consistent with the spectro-temporal scales of rat ultrasonic vocalizations. Simulations and SignalTap analyses confirmed that this setting was adequate to resolve both 22 kHz and 50 kHz calls. Hardware tests were performed by injecting sinusoidal signals into the analog front-end, digitizing them on the FPGA, and exporting FFT results through SignalTap. The resulting spectra consistently showed clear peaks at the input frequency, and multiple frames demonstrated stable repetition over time, validating both the FFT computation and its temporal continuity (Fig. 12).

**Fig. 13:**
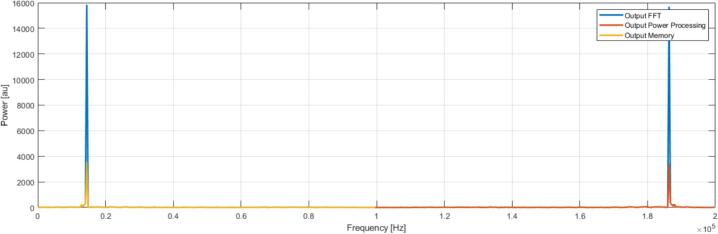
Graph showing the direct output of the FFT (blue), the output after power processing (red), and the final output after transmitting only half of the spectrum (yellow). (For interpretation of the references to colour in this figure legend, the reader is referred to the web version of this article.)

Post-processing of the FFT outputs on the FPGA included real-time calculation of the signal power from the real and imaginary components, followed by a memory stage that retained only the first 256 points of each frame. This ensured that only the non-redundant half of the spectrum was transferred to the computer, thereby reducing bandwidth while preserving all relevant information. SignalTap measurements and Matlab reconstructions confirmed that the computed power spectra matched expectations, with the correct frequency peaks preserved after hardware power calculation and memory sorting (Fig. 13).

**Fig. 14:**
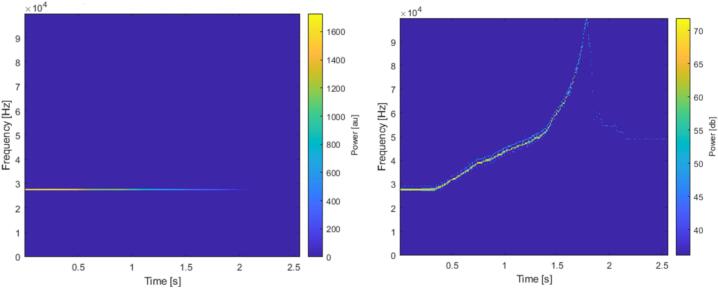
Spectrogram showing the variation of the amplitude (on the left) and the frequency (on the right) of the input signal.

Spectrograms reconstructed from FPGA outputs showed the expected time–frequency structure of sinusoidal test signals, validating the complete acquisition and processing chain (Fig. 14). While the absolute amplitude of the FFT peaks depended on which subset of bits was selected from the 24-bit power calculation, the overall shape and frequency placement of the peaks remained consistent.

Capabilities and limitations of the FPGA stage:

• Correct acquisition of analog signals by the on-board 12-bit ADC, validated by SignalTap monitoring.
• FFT processing at 512 points confirmed, yielding ∼390 Hz frequency resolution and ∼2.56 ms time resolution.
• Hardware power calculation and memory stage validated, preserving spectral content while halving data volume.
• Spectrograms reconstructed from FPGA outputs consistent with injected test signals.
• Validation of each design stage performed with testbench simulations and/or SignalTap debugging; detailed results not included here for brevity.

### 9.4 Software

Validation of the computer software focused on verifying its ability to receive, store, and display in real time the spectral data transmitted by the FPGA. The application, implemented in C with GTK4, provides three main functionalities: initiating and stopping data acquisition, recording streamed FFT frames into a file, and rendering an online spectrogram for visual inspection during experiments.

Tests of serial communication confirmed that the software could reliably handle the 3 Mbaud data stream produced by the FPGA via the FTDI interface. Frame boundaries, identified by dedicated header bits, were correctly detected by the program, and no major data corruption was observed during extended transfers. However, because UART is not a protocol with built-in error detection or retransmission, rare frame losses were observed (on the order of one missing byte per million transmitted). These events had no impact on the continuity of the spectrogram or on the ability to detect ultrasonic vocalizations, but they illustrate the inherent limitation of the communication protocol.

The recording function was validated by saving multiple sessions into CSV files. Offline inspection of these files confirmed that the format was consistent and directly usable for further analysis. Spectrograms reconstructed from the recorded files matched the real-time display, demonstrating that no data loss occurred between acquisition, visualization, and storage beyond the rare UART-level frame slips mentioned above.

The GTK-based graphical interface was tested during acquisition of synthetic ultrasonic signals injected at different frequencies. The online display showed the expected time–frequency structure, with stable axis scaling and correct color mapping of spectral intensity. The refresh rate was sufficient to follow dynamic changes in the input signal, validating the usability of the software for live monitoring.

Capabilities and limitations of the computer software:

• Reliable reception of FFT frames at 3 Mbaud.
• Correct parsing of frame headers and formatting into usable spectra.
• Real-time spectrogram rendering with stable axes and correct intensity mapping.
• Recording data in CSV format.
• Synchronization between live display and stored data, ensuring consistency.
• Very rare frame losses (≈ 1 byte per 10⁶ transmitted) due to inherent limitations of UART.

### 9.5 Full system validation in real conditions

The complete OCTAVie system was validated in real experimental conditions by recording USVs emitted by paired-housed young adult Long Evans rats (Janvier Labs, Le Genest-Saint-Isle, France) during rough-and-tumble play in their home cage. In this configuration, the chain integrated all subsystems − microphone, analog anti-aliasing filter, FPGA acquisition and FFT, and computer software − operating together as in a typical neuroscientific setting. The microphone was positioned approximately 5 cm above the cage floor, and recordings were performed at a sampling rate of 200 kS/s using the 512-point FFT configuration described above. Each session lasted approximately 30 min.

**Fig. 15:**
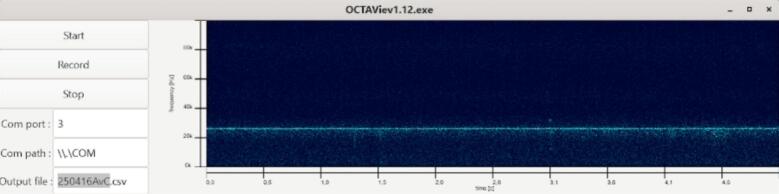
Background noise recorded in the experimental room.

**Fig. 16:**
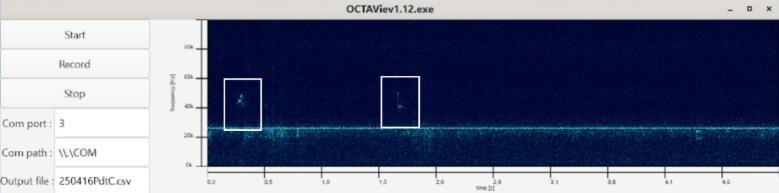
50 kHz vocalization recorded.

Recordings performed during behavioral protocols produced spectrograms in which vocalizations were clearly identifiable. Calls were defined as time–frequency structures within the 18–80 kHz band exhibiting a signal-to-noise ratio visibly exceeding background fluctuations and durations consistent with previously described rat USVs. Calls were detected with temporal and spectral characteristics consistent with those associated with a positive emotional state as described in the literature [3], [4], [5], [6]. The online graphical interface displayed these calls in real time, while the recorded CSV files allowed offline inspection and reproduction of the spectrograms. The recordings presented here were performed in a standard laboratory room with noticeable background noise caused by the ventilation system (Fig. 15). For optimal results, we recommend conducting experiments in a soundproof or acoustically treated environment. However, due to limited resources, our tests were carried out in a noisy setting, which further demonstrates the robustness and reliability of the OCTAVie system under non-ideal acoustic conditions since vocalizations were observable (Fig. 16).

Overall, these results demonstrate that the system meets its primary scientific objective: enabling the detection and visualization of ultrasonic rat vocalizations at low cost and with open-source hardware and software. Although the absolute acoustic amplitude of the signals was not calibrated, the presence, timing, and frequency content of the calls were reliably captured. The spectrograms produced were sufficiently resolved to distinguish different call categories, confirming that the design is appropriate for ethological and behavioral studies.

## Ethics statements

Animal housing conditions and care were in compliance with the European Community Council Directive (2010/63/UE). The home-cage recording of vocalization was made in a group of female rats included in a project approved by the regional ethical committee and the French Ministry of National Education, Higher Education and Research (APAFIS#44368).

## CRediT authorship contribution statement

**Laura Durieux:** Writing – review & editing, Writing – original draft, Validation, Supervision, Software, Resources, Methodology, Investigation, Formal analysis, Conceptualization. **Jean-Baptiste Kammerer:** Writing – review & editing, Writing – original draft, Validation, Software, Resources, Investigation, Conceptualization. **Simon Schmitt:** Validation, Software, Resources, Methodology, Investigation, Conceptualization. **Denis Muller:** Validation, Resources, Investigation. **Alexandra Barbelivien:** Writing – review & editing, Validation, Resources, Methodology, Formal analysis. **Monique Majchrzak:** Writing – review & editing, Validation, Resources, Methodology, Formal analysis. **François Stock:** Writing – review & editing, Validation, Supervision, Software, Resources, Methodology, Investigation, Conceptualization. **Frederic Antoni:** Writing – review & editing, Validation, Supervision, Resources, Project administration, Methodology, Investigation, Funding acquisition, Conceptualization.

## Funding

This work was supported by ICube Laboratory through internal funding (API).

## Declaration of competing interest

The authors declare the following financial interests/personal relationships which may be considered as potential competing interests: Frederic ANTONI reports administrative support and article publishing charges were provided by Laboratoire des Sciences de l’Ingénieur de l’Informatique et de l’Imagerie. If there are other authors, they declare that they have no known competing financial interests or personal relationships that could have appeared to influence the work reported in this paper.
